# BrazIliaN Type 1 & 2 DiabetEs Disease Registry (BINDER): longitudinal, real-world study of diabetes mellitus control in Brazil

**DOI:** 10.3389/fcdhc.2022.934629

**Published:** 2022-08-16

**Authors:** Bianca de Almeida-Pititto, Freddy G. Eliaschewitz, Mauricio A. de Paula, Graziela C. Ferreira

**Affiliations:** ^1^Escola Paulista de Medicina, Universidade Federal de São Paulo, São Paulo, Brazil; ^2^Centro de Pesquisas Clinicas (CPCLIN)/Diagnósticos da América S.A (DASA), Centro de Pesquisas Clínicas, São Paulo, Brazil; ^3^Sanofi, São Paulo, Brazil

**Keywords:** Diabetes mellitus, type 1, Diabetes mellitus, type 2, real-world, HbA1c, glycemic

## Abstract

**Introduction:**

This study aimed at assessing the patterns of care and glycemic control of patients with diabetes (DM) in real life during a follow-up of 2 years in the public and private health sectors in Brazil.

**Methods:**

BINDER was an observational study of patients >18 years old, with type-1 (T1DM) and type-2 DM (T2DM), followed at 250 sites from 40 cities across the five regions of Brazil. The results for the 1,266 participants who were followed for 2 years are presented.

**Main results:**

Most patients were Caucasians (75%), male (56.7%) and from the private health sector (71%). Of the 1,266 patients who entered the analysis, 104 (8.2%) had T1DM and 1162 (91.8%) had T2DM. Patients followed in the private sector represented 48% of the patients with T1DM and 73% of those with T2DM. For T1DM, in addition to insulins (NPH in 24%, regular in 11%, long-acting analogues in 58%, fast-acting analogues in 53%, and others in 12%), the patients received biguanide (20%), SGLT2-I (4%), and GLP-1Ra (<1%). After 2 years, 13% of T1DM patients were using biguanide, 9% SGLT2-I, 1% GLP-1Ra, and 1% pioglitazone; the use of NPH and regular insulins decreased to 13% and 8%, respectively, while 72% were receiving long-acting insulin analogues, and 78% fast-acting insulin analogues. Treatment for T2DM consisted of biguanide (77%), sulfonylureas (33%), DPP4 inhibitors (24%), SGLT2-I (13%), GLP-1Ra (2.5%), and insulin (27%), with percentages not changing during follow-up. Regarding glucose control, mean HbA1c at baseline and after 2 years of follow-up was 8.2 (1.6)% and 7.5 (1.6)% for T1DM, and 8.4 (1.9)% and 7.2 (1.3)% for T2DM, respectively. After 2 years, HbA1c<7% was reached in 25% of T1DM and 55% of T2DM patients from private institutions and in 20.5% of T1DM and 47% of T2DM from public institutions.

**Conclusion:**

Most patients did not reach the HbA1c target in private or public health systems. At the 2-year follow-up, there were no significant improvements in HbA1c in either T1DM or T2DM, which suggests an important clinical inertia.

## Introduction

According to the International Diabetes Federation, 463 million people are currently living with diabetes (DM) worldwide (1, 2). In 2019, it was estimated that there were about 16.8 million people aged from 20 to 79 years with DM in Brazil, with a projected increase of 55% by the year 2045 (1, 2). Type 2 diabetes (T2DM) comprises approximately 90% of all DM diagnoses (3). Estimates related to the number of existing cases of type 1 diabetes (T1DM) in children and adolescents from 0 to 14 years show that Brazil occupies the third place in the global panorama, with 55,500 cases, behind India (95,600) and the United States (94,200) (1).

Chronic non-communicable diseases (NCDs) are responsible for nearly two thirds of deaths in Brazil, 5.3% of which due to DM (4). In addition, DM is known to be an important risk factor for chronic cardiovascular disease (CVD), which accounts for 31.3% of deaths in our country (5).

Over the last decades, age-standardized rates have shown a tendency to reduced mortality caused by CVD and DM in Brazil (6, 7), in agreement with the aging of the population and the extension of life with the disease. The considerable burden of these diseases was highlighted in the Project on the Global Burden of Disease in Brazil (Burden of disease in Brazil, 1990–2016), in which DM was identified to be responsible for 4.7% of disability-adjusted life-years (DALY) in total and 6.1% of DALY originated by NCDs (8).

One of the great current challenges is, therefore, to deal with this increase in morbidity, which requires controlling the disease and preventing complications. These data are even more worrisome when considering the number of affected people in Brazil. Brazilian data on the prevalence of DM representative of the population of nine capitals date from the 1980s (9). At that time, it was estimated that approximately 7.6% of the Brazilian population aged between 30 and 69 years had DM, with both genders being equally affected, and with the prevalence of the disease increasing with age and body fat. A more recent estimate of the prevalence of self-reported DM in Brazil was performed by the Surveillance System of Risk and Protective Factors for Chronic Diseases by Telephone Survey (VIGITEL, *Vigilância de Fatores de Risco por Inquérito Telefônico*), implemented in 27 state capitals since 2006 (10). In the VIGITEL 2018, 8.1% of women and 7.1% of men ≥18 years old in Brazil reported having DM; the numbers increased with age, reaching 23.1% in individuals over 64 years of age, and decreased with higher the level of education, affecting 15.2% of the participants with from 0 to 8 years of schooling and 3.7% in the group with higher education (10).

The high prevalence of DM exerts a negative impact on health not only due to mortality, but also through complications and disabilities resulting from the prolonged time living with the disease and poor metabolic control. In addition to the health-related effects, diabetes is associated with an unwanted economic impact on both individual and society levels. Studies show that associated costs increase according to the duration of DM and the presence of micro- and macrovascular complications (11, 12). Inadequate glycemic control can aggravate these medical conditions and has been reported in studies including patients with T1DM and T2DM treated in the Brazilian Public Unified Health System (SUS, *Sistema Único de Saúde*) (11, 13, 14). Data related to the management of diabetes in the private sector in Brazil are still scarce.

To understand this scenario, there is a lack of data on the prevalence of chronic complications and comorbidities, including cardiovascular risk factors, associated with DM in the Brazilian population. In this regard, public and private health services represent opportunities to access professional care and different medications, providing information to guide better strategies for secondary and tertiary prevention of DM. The disease burden of DM is a relevant concern that requires secondary and tertiary prevention strategies. To develop these actions, it is necessary to understand the epidemiological and current management landscape of patients with diabetes in Brazil.

The BrazIliaN Type 1 & 2 DiabetEs Disease Registry (BINDER) study was an observational study, with both a cross-sectional and a longitudinal phase, designed to assess the demographic and clinical characteristics, patterns of care and glycemic control of patients with DM in real life during a follow-up of 2 years in the public and private health sectors in Brazil. In this paper, we present the results of the longitudinal analysis which included the patients followed for 2 years.

## Patients and methods

### Study design and population

This was a observational study of individuals with DM followed for 2 years in the BINDER study. BINDER included patients with T1DM and T2DM followed by 250 physicians from different public and private healthcare services, geographically distributed in 40 cities across the five regions of Brazil. The study had both cross-sectional and longitudinal phases (for a total duration of 2 years). Five waves of data collection were performed; for each wave, information from the last 6 months was obtained. To be enrolled in the study, patients had to be 18 years or older, have T1DM or T2DM, and had to have attended at least one medical visit at the study site in the 6 months prior to study entry. Pregnancy, gestational diabetes and other types of DM except T1DM or T2DM were excluded.

Each medical specialist (endocrinologists, cardiologists, or general practitioners) was responsible for recruiting about ten patients. To minimize patient selection bias, investigators were instructed to recruit patients in a retrospective consecutive manner starting from the patients that were last seen in the service according to medical charts. The initial sample of the study comprised 2,488 patients who entered the first wave of data collection (baseline visit). In the longitudinal phase, four subsequent follow-up visits were planned to occur every 6 months until the completion of the 2-year follow-up period. In this paper, we present the results obtained for the 1,266 participants who completed the final visit scheduled to occur after 2 years of follow-up and comprised the population of the longitudinal analysis.

Participating study centers were selected by the Associação Brasileira de Organizações Representativas de Pesquisa Clínica according to a proprietary database. A total of 250 sites/medical specialists of 40 Brazilian cities of the five country regions were chosen: 124 in the Southeast; 48 in the Northeast; 38 in the South Region; 30 in the Central-West Region; and 10 in the North Region.

The participant physicians collected data from patient medical charts covering the medical appointments that occurred from 07-Apr-2016, the date of study initiation, to 13-Dec-2019, the date of the final visit for the study.

The study was conducted after the approval by the ethics committee of the Universidade Federal de São Paulo (São Paulo, Brazil), and the study was conducted in accordance with the Declaration of Helsinki and the International Conference on Harmonization guidelines for Good Clinical Practice. Informed consent was obtained from all patients.

### Data collection, variables and evaluation criteria

Data were collected from medical charts using an electronic CRF (e-CRF), and data management was performed according to the Data Validation Plan with data review processes in order to clarify data issues.

Variables of interest in the cross-sectional (baseline) phase were age, gender, ethnicity, educational level, body mass index (BMI), age at diagnosis, DM duration (time since diagnosis), abdominal circumference, blood pressure and laboratory results, risk factors for CVD, comorbidities, DM complications, glycemic control, medical specialties involved in patient care, and type of treatment. For the subsequent waves and longitudinal phase, collected data included glycemic control (HbA1c), weight, BMI, use of insulins and other medications, number of medications, and comorbidities and complications.

The achievement of individual HbA1c target (<7.0% or defined individual target) in patients with T1DM and T2DM at the study baseline (cross-sectional phase) and after 2 years of follow-up was the primary objective of the study and was described by the proportion of patients who reached the target in the overall study population and per DM type. The proportion was complemented by the respective 95% confidence interval (CI). Secondary objectives included the description of patients regarding their demographic and clinical characteristics, presence of comorbidities, complications, patterns of treatment and hospitalizations at baseline and during the follow-up period.

As this is a disease registry, non-interventional study, no data were collected beyond those required for routine clinical practice. However, Adverse Drug Reactions to any Sanofi product that occurred during the course of the study was to be reported to the Sponsor within 24 hours from the moment the investigator was notified about the case, in compliance with pharmacovigilance practice.

### Statistical considerations and analysis

Statistical analysis was based on pooled data from all patients. Given the observational nature of the study, the statistical analysis was mainly descriptive, using appropriate summary statistics according to the type of variable. Descriptive statistics as number of non-missing data, range (minimum and maximum values), mean, standard deviation (SD), median and interquartile range (IQR) were calculated for summarizing numerical variables. Frequencies and proportions were calculated for summarizing categorical variables. There was no data imputation for missing/not available data in the calculations. The number of participants with available information for each variable are displayed in the tables, when considered relevant.

For the longitudinal phase, statistical analysis was based on pooled data of all patients who had available data at baseline and also at the end of follow-up, after 2 years. Descriptive analyses were performed according to the DM type and health care system (private and public sectors).

For the cross-sectional phase, nearly 2,500 patients were planned to be enrolled. Considering a planned sample size of 2,500 patients for the cross-sectional phase and assuming that T2DM comprise 90% of DM cases, the study expected to recruit about 2,250 patients with T2DM and 250 patients with T1DM. The sample size of 2,250 T2DM patients would ensure 95% CIs with a maximum width of 2.1% below and above point estimate. On the other hand, with a sample size of 250 T1DM patients, the maximum expected width was 6.2% below and above the point estimate.

Sample size calculation was performed based on published data from population studies conducted in Brazil that estimated the proportion of patients with HbA1c values within the target. Considering an expected proportion of 27% of patients with T2DM within the HbA1c target (3), the sample of 2,250 patients with T2DM would allow assessing this proportion with 95% CIs with a maximum width of 1.8% below and above the point estimate; and for an expected proportion of 10% of T1DM patients within the HbA1c target (14), the sample of 250 patients with T1DM would allow assessing this proportion with 95% CIs with a maximum width of 3.7% below and above the point estimate.

## Results

Baseline characteristics and comorbidities of the subset of patients who entered the longitudinal analysis were similar to those of the patients comprising the total study sample (data not shown). The baseline sample comprised 91.9% of patients with T2DM, the mean age was 63 years, and 52.2% were from the Southeast Region, while the sample at the end of the follow-up period had 91.8% of patients with T2DM, mean age of 62 years, and 51.8% from the Southeast Region.

### Patient characteristics

The study sample for the longitudinal analysis comprised a total of 1,266 patients of the BINDER study who had completed the 2 years of follow-up with data collection in all five waves. As shown in **Table 1**, 56.7% of patients were male, 74.7% were Caucasian, and 33.5% had a college or higher degree of education. One hundred and four patients had T1DM (8.2%), and 1162 (91.8%) had T2DM. At the time of the initial study visit, the mean age of T1DM and T2DM patients were 35.0 and 63.7 years, respectively; patients aged 18 to 30 years comprised 38.5% of the T1DM group and 0.5% of the T2DM patients. T1DM patients were under treatment for a longer time (mean treatment duration: 15.8years for T1DM *vs* 9.8 years for T2DM), although the mean time since DM diagnosis was similar between T1DM (16.5 years) and T2DM (17.8 years). Of the assessed patients, 48% of those with T1DM and 73.2% of those with T2DM were followed in the private health sector (**Table 1**). A family history of DM was reported by 12.2% and 25.3% of patients with T1DM and T2DM, respectively.

**Table 1:** Demographic and clinical characteristics.

Characteristic	All Patients n=1266	T1DM n=104	T2DM n=1162
**Gender, n (%)**	*n=1263*	*n=104*	*n=1159*
Male	716 (56.7%)	65 (62.5%)	651 (56.2%)
Female	547 (43.3%)	39 (37.5%)	508 (43.8%)
**Age, years**	*n=1262*	*n=104*	*n=1158*
Mean ± SD	61.3 ± 14.3	35.0 ± 12.0	63.7 ± 11.9
Range	18 – 93	18 – 74	19 – 93
**Age, years**	*n=1262*	*n=104*	*n=1158*
18 to 30 years	46 (3.6%)	40 (38.5%)	6 (0.5%)
31 to 50 years	193 (15.3%)	50 (48.1%)	143 (12.3%)
51 to 70 years	680 (53.9%)	13 (12.5%)	667 (57.6%)
>70 years	343 (27.2%)	1 (1.0%)	342 (29.5%)
**Ethnicity**, **n (%)**	*n=997*	*n=90*	*n=907*
Caucasian	745 (74.7%)	67 (74.4%)	678 (74.8%)
Brown/Latin/Mixed	117 (11.7%)	19 (21.1%)	98 (10.8%)
Black	107 (10.7%)	4 (4.4%)	103 (11.4%)
Asian/Indigenous/Yellow	28 (2.8%)	0 (0%)	28 (3.1%)
**Country region**	*n=1266*	*n=104*	*n=1162*
Southeast	656 (51.8%)	34 (32.7%)	622 (53.5%)
South	208 (16.4%)	17 (16.3%)	191 (16.4%)
Northeast	146 (11.5%)	34 (32.7%)	112 (9.6%)
Central-West	181 (14.3%)	19 (18.3%)	162 (13.9%)
North	75 (5.9%)	0 (0.0%)	75 (6.5%)
**Formal education**	*804*	*80*	*724*
College or higher	269 (33.5%)	36 (45.0%)	233 (32.2%)
High school	291 (36.2%)	31 (38.8%)	260 (35.9%)
Elementary school	227 (28.2%)	13 (16.2%)	214 (29.6%)
Illiterate	17 (2.1%)	0	17 (2.3%)
**Medical specialty**	*n=1266*	*n=104*	*n=1162*
Endocrinologist	620 (49%)	81 (77.9%)	539 (46.4%)
General Practitioner	241 (19%)	16 (15.4%)	225 (19.4%)
Cardiologist	405 (32%)	7 (6.7%)	398 (34.3%)
**Health-care sector**	*n=1208*	*n=100*	*n=1108*
Private	859 (71.1%)	48 (48.0%)	811 (73.2%)
Public	349 (28.9%)	52 (52.0%)	297 (26.8%)
**Family history of diabetes**	297 (24.4%)	11 (12.2%)	286 (25.3%)
**Age at first diagnosis, years**	*n=1104*	*n=101*	*n=1003*
Range	1 to 88	3 to 70	1 to 88
Mean ± SD	49.9 ± 16	19 ± 11.6	53 ± 12.7
Median (IQR)	52 (1 – 88)	17 (12 – 24)	54 (45 – 61)
**Time since first diagnosis, years**	*n=1266*	*n=104*	*n=1162*
Range	0 to 92	0 to 45	0 to 92
Mean ± SD	17.7 ± 20.2	16.5 ± 10.4	17.8 ± 20.9
Median (IQR)	10 (5 – 20)	15 (8 – 23.5)	10 (4 – 20)
**Treatment duration, years**	*n=1014*	*n=92*	*n=922*
Range	0 to 70	2 to 45	0 to 70
Mean ± SD	10.3 ± 8.4	15.8 ± 9.7	9.8 ± 8.1
Median (IQR)	8 (4 – 15)	14 (8 – 23)	7 (4 – 14)

IQR, interquartile range (25^th^ – 75^th^); n, number of patients with available information; SD, standard deviation.

Of the 104 patients with T1DM, 77.9% were followed by endocrinologists, 15.4% by general practitioners, and 6.7% by cardiologists, while among those with T2DM, 46.4% were followed by endocrinologists, 19.4% by general practitioners, and 34.2% by cardiologists.

Overall, the number of medical appointments for DM management per year ranged from 1 to 21. The median (IQR) number of consultations per year were 2 (1-4) and 1 (1-3) for T1DM and T2DM, respectively.

### Comorbidities and complications associated with DM

Considering the information collected from baseline until the end of the 2-year follow-up, 1,219 (96.3%) patients presented at least one comorbidity or complication associated with DM (**Table 2**). Patients with T1DM presented a lower prevalence of hypertension (31.1% vs 82.4%), dyslipidemia (48.9% vs 77.9%), overweight/obesity (22.2% vs 40.7%) and smoking habit (2.2% vs 8.8%) and a higheprevalence of hypothyroidism (33.3% vs 15.0%) than those with T2DM. Similar frequencies of sedentarism, elevated uric acid and sleep apnea were observed between groups.

**Table 2:** Comorbidities and complications related to diabetes during the study period.

	All Patients n=1266	T1DM n=104	T2DM n=1162
Medical conditions related to diabetes*			
None	47 (3.7%)	14 (13.5%)	33 (2.8%)
Any (at least one)	1219 (96.3%)	90 (86.5%)	1129 (97.2%)
**Comorbidities**			
Hypertension	958 (78.6%)	28 (31.1%)	930 (82.4%)
Dyslipidemia	923 (75.7%)	44 (48.9%)	879 (77.9%)
Obesity/overweight	479 (39.3%)	20 (22.2%)	459 (40.7%)
Sedentary life	379 (31.1%)	25 (27.8%)	354 (31.4%)
Hypothyroidism	199 (16.3%)	30 (33.3%)	169 (15.0%)
Smoking	101 (8.3%)	2 (2.2%)	99 (8.8%)
Elevated uric acid	53 (4.3%)	3 (3.3%)	50 (4.4%)
Sleep apnea	36 (3.0%)	3 (3.3%)	33 (2.9%)
**Complications**			
Cardiovascular disease	269 (22.1%)	3 (3.3%)	266 (23.6%)
History of infarction	152 (12.5%)	4 (4.4%)	148 (13.1%)
Other coronary diseases	167 (13.7%)	5 (5.6%)	162 (14.3%)
Retinopathy	161 (13.2%)	35 (38.9%)	126 (11.2%)
Neuropathic pain	132 (10.8%)	22 (24.4%)	110 (9.7%)
Renal complications/renal failure	122 (10%)	18 (20.0%)	104 (9.2%)
Microalbuminuria	128 (10.5%)	19 (21.1%)	109 (9.7%)
Stroke history	71 (5.8%)	0	71 (6.3%)
Diabetic foot	31 (2.5%)	8 (8.9%)	23 (2.0%)
Impotence	32 (2.6%)	1 (1.1%)	31 (2.7%)
Unstable angina	17 (1.4%)	2 (2.2%)	15 (1.3%)
Blindness	14 (1.1%)	3 (3.3%)	11 (1.0%)
Amputation of limbs	12 (1.0%)	1 (1.1%)	11 (1.0%)

n, number of patients with available information. Other medical conditions reported in the study, but not included in this table due to their lower frequency were hyperthyroidism, skin problems in general, gout, pancreatic disorder, organ disorder, organ transplantation, and tuberculosis.

Regarding chronic complications, microvascular complications (retinopathy, blindness, microalbuminuria, and renal disease), diabetic foot and neuropathy were more prevalent in patients with T1DM, while macrovascular complications (CVD, history of infarction, other coronary diseases, and stoke history) and erectile dysfunction were more frequently reported in the group of T2DM. The prevalence of lower-limb amputation did not differ between groups.

Dyslipidemia treatment since the baseline assessment reached the frequency of 97% in both groups (not shown).

Regarding the medications used for treatment of DM at the final visit, as shown in **Table 3**, after 2 years 100% of T1DM patients were using insulin, together with oral medications such as biguanide (13.0%), iSGLT2 (8.7%), and pioglitazone (1.1%), or with non-insulin injectable medications such as GLP1 (1.1%). Patients with T2DM used oral medications at a higher frequency: biguanide (70.4%), sulfonylurea (31.4%), DPP4 inhibitor (26.8%), SGLT2 inhibitor (21.9%) and pioglitazone (4.4%); regarding injectables, 2.0% were in use of GLP1 and 27.3% were in use of a type of insulin.

**Table 3:** Diabetes treatments in use at baseline and at the final follow-up.

	T1DM		T2DM	
DM treatment	Baseline	Final	Baseline	Final
	*n=104*	*n=92*	*n=1115*	*n=990*
Biguanide (Metformin)	21 (20.2%)	12 (13.0%)	862 (77.3%)	697 (70.4%)
Sulfonylurea	1 (1.0%)	0 (0%)	371 (33.3%)	311 (31.4%)
DPP4 Inhibitor	0	0 (0%)	270 (24.2%)	265 (26.8%)
SGLT2 Inhibitor	4 (3.8%)	8 (8.7%)	145 (13%)	217 (21.9%)
GLP-1	0	1 (1.1%)	28 (2.5%)	20 (2.0%)
TZD (Pioglitazone)	0	1 (1.1%)	0	44 (4.4%)
Apha-Glucosidade Inhibitor (Acarbose)	0	0 (0%)	0	3 (0.3%)
Glinides	0	0 (0%)	0	1 (0.1%)
Insulin	103 (99.0%)	92 (100%)	302 (27.1%)	270 (27.3%)
NPH	25 (24%)	12 (13.0%)	191 (17.1%)	172 (17.4%)
Long-acting insulin analogues	60 (57.7%)	66 (71.7%)	90 (8.1%)	81 (8.2%)
Fast-acting insulin analogues	55 (52.9%)	72 (78.3%)	38 (3.4%)	40 (4.0%)
Regular	11 (10.6%)	7 (7.6%)	44 (3.9%)	47 (4.7%)
Premixed	1 (1%)	5 (5.4%)	18 (1.6%)	13 (1.3%)
Other insulins	12 (11.5%)	0 (0%)	3 (0.3%)	2 (0.2%)
Combined	0	1 (1.1%)	0	17 (1.7%)

It is interesting to note that 28 (26.9%) T1DM patients and 280 (24.3%) T2DM patients reported having discontinued the medication in some moment during the study (**Table 4**). The main reasons mentioned for drug withdrawal were lack of efficacy (28.6%), hypoglycemia risk (17.9%), and difficulty in dose titration (17.9%) for T1DM patients; while for patients with T2DM these were lack of efficacy (27.5%), hypoglycemia risk (18.2%), and cost (16.1%).

**Table 4:** Reported reasons for treatment discontinuation, according to the type of DM.

	T1DM	T2DM
**Patients using any drug**	*n=104*	*n=1153*
**Patients with any withdrawal reported, n (%)**	28 (26.9%)	280 (24.3%)
**Reason for discontinuation, n (%)**	*N=28*	*N=280*
Lack of efficacy	8 (28.6%)	77 (27.5%)
Hypoglycemia risk	5 (17.9%)	51 (18.2%)
Difficulty in dose titration	5 (17.9%)	16 (5.7%)
Patient request	2 (7.1%)	37 (13.2%)
Cost	0 (0.0%)	45 (16.1%)
Adverse events	1 (3.6%)	25 (8.9%)
Route of administration	1 (3.6%)	9 (3.2%)
Drug interaction	1 (3.6%)	8 (2.9%)
Other reason	13 (46.4%)	111 (39.6%)

### Glycemic control

**Figure 1:**
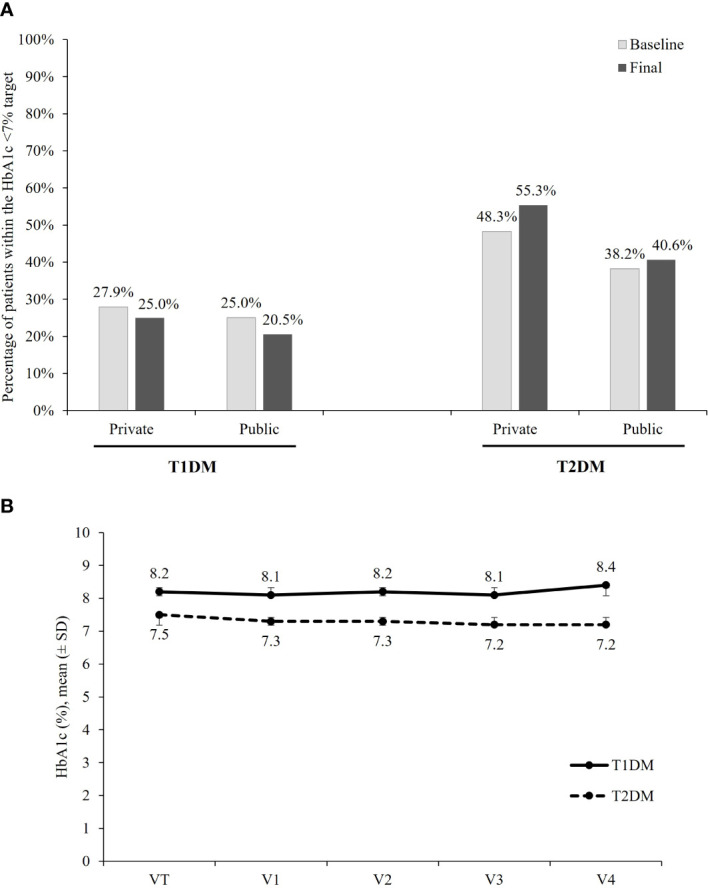
HbA1c. **(A)** Percentage of patients achieving the target of HbA1c <7%, according to the DM type and health care sector. **(B)** Mean ( ± SD) HbA1c (%) per visit, according to the DM type.

**Figure 1A** shows the percentage of patients within the target HbA1c <7% at baseline and at the last follow-up visit, according to the type of DM and health service. At the baseline assessment, 22 (25.3%; 95% CI, 16.2% to 34.4%) T1DM patients had HbA1c levels <7% and 23 (29.5%; 95% CI, 19.4% to 39.6%) were within the individual goal. After 2 years of follow-up, the percentages of patients with HbA1c <7% and HbA1c within the individual target were 22.5% (n=18; 95% CI, 13.4% to 31.6%) and 27.8% (n=20; 95% CI, 17.4% to 38.1%), respectively. For T2DM patients, the proportion of patients within the goal of HbA1c <7% changed from 45.5% (n=375; 95% CI, 42.1% to 48.9%) at baseline to 51.0% (n=369; 95% CI, 47.4% to 54.7%) after 2 years follow-up, while the proportion of patients within the individual HbA1c target changed from 47.1% (n=364; 95% CI, 43.6% to 50.6%) to 54.4% (n=356; 95% CI, 50.6% to 58.2%).

The analysis according to health-care system showed that after 2 years, the target of HbA1c <7% was reached in 25.5% (95% CI, 10.9% to 39.1%) of T1DM and in 55.3% (95% CI, 51.1% to 59.5%) of T2DM patients being followed in the private sector and in 20.5% (95% CI, 8.5% to 32.4%) of T1DM and 40.6% (95% CI, 33.4% to 47.7%) of T2DM treated in the public sector. For patients with T1DM, no difference in the proportion of patients within the goal was observed between patients followed in the public and in the private sectors. For patients with T2DM, results suggest a better glycemic control in the private sector at baseline as well as after 2 years of follow-up.

**Figure 1B** shows the mean (± SD) HbA1c in each of the five waves of data collection, according to the type of DM. The mean ± SD of HbA1c at baseline and after 2 years of follow-up was, respectively, 8.2 (1.6)% and 8.4 (1.9)% among T1DM patients, and 7.5 (1.6)% and 7.2 (1.3)% among those with T2DM.

### Weight control

**Table 5** shows the changes in BMI from baseline to the final visit. In both groups, the mean change in BMI was low (0 ± 2.4 Kg/m^2^ in T1DM, and -0.3 ± 2.2 Kg/m^2^ in T2DM). After 2 years, most patients had maintained their weight and only a minority of patients had a decrease in the BMI category.

**Table 5:** Changes in BMI from baseline to the final follow-up (after 2 years).

	All patients	T1DM	T2DM
**Change in BMI from baseline to the final visit, in Kg/m^2^**	*n=749*	*n=84*	*n=665*
Mean ± SD	-0.2 ± 2.3	0 ± 2.4	-0.3 ± 2.2
Median (IQR)	0 (-1 – 0.7)	0.3 (-0.8 – 1)	0 (-1.1 – 0.7)
**BMI category (Kg/m²) in the final visit in relation to baseline, n (%)**	*n=749*	*n=84*	*n=665*
No change from baseline to the final visit	612 (81.7%)	71 (84.5%)	541 (81.3%)
Increase in the BMI category from baseline to the final visit	56 (7.5%)	9 (10.7%)	47 (7.1%)
≤24.9 to ≥25–29.9 Kg/m²	22 (2.9%)	6 (7.1%)	16 (2.4%)
≤24.9 to ≥30–39 Kg/m²	2 (0.3%)	0	2 (0.3%)
25–29.9 to ≥30–39 Kg/m²	23 (3.1%)	3 (3.6%)	20 (3.0%)
≥30–39 to >39.9 Kg/m²	9 (1.2%)	0	9 (1.4%)
Decrease in BMI category from baseline to the final visit	81 (10.8%)	4 (4.8%)	77 (11.6%)
25–29.9 Kg/m² to ≤24.9 Kg/m²	34 (4.5%)	2 (2.4%)	32 (4.8%)
30–39.9 Kg/m² to 25–29.9 Kg/m²	36 (4.8%)	2 (2.4%)	34 (5.1%)
30–39.9 Kg/m² to ≤24.9 Kg/m²	2 (0.3%)	0	2 (0.3%)
>39.9 Kg/m² to 30–39.9 Kg/m²	7 (0.9%)	0	7 (1.1%)
>39.9 Kg/m² to 25–29.9 Kg/m²	1 (0.1%)	0	1 (0.2%)
>39.9 Kg/m² to Up to 24.9 Kg/m²	1 (0.1%)	0	1 (0.2%)

## Discussion

The BINDER study represented an important opportunity to observe in a real-word scenario the patterns of disease management, glycemic control, DM-associated complications and morbidities of patients with T1DM and T2DM for a period of 2 years of follow-up in the public and private health sectors in Brazil.

In relation to the sociodemographic profile of participants and distribution of care, the sample population was not intended to represent the Brazilian population; as a result, the study sample comprised 74.7% of Caucasians and 33.5% of patients having a high level of education, which are above nationwide proportions. However, it is important to emphasize that, from the point of view of the distribution of DM types, we found a distribution similar to the one reported in large epidemiological studies, with T2DM accounting for about 90% of patients (1). In the current study, of the 1,266 patients followed for 2 years, 73.2% of T2DM and 48% of T1DM patients were seen in the private health sector, thus offering an opportunity to assess the profile of morbidities and glycemic control in this kind of health-care service. Of note, patients with T1DM were more commonly seen in the public healthcare system than T2DM patients, which may be indicative of a greater preparation and availability of drugs for the management of T1DM in specialized public services, such as tertiary services and centers linked to Universities. In addition, we also observed that patients with T1DM were seen in the vast majority of cases (77.9%) by endocrinologists, while the management of patients with T2DM was more distributed between medical specialties, with nearly 50% of cases being seen by endocrinologists, followed by general practitioners (19.4%) and cardiologists (34.2%). This result contrasts with a previous study in which it was found that in the public service, nearly 80% of patients with T2DM were followed by a general practitioner (10).

Nearly 97% of patients presented at least one associated morbidity, with this percentage being higher among patients with T2DM than in those with T1DM (97.2% vs. 86.5%). Of note, T2DM patients had a mean age higher than 60 years at the baseline assessment. This high prevalence of associated morbidities is in agreement with estimates reported in health surveys and epidemiological studies conducted in the elderly population in Brazil. Results from the Brazilian National Health Survey (PNS) of 2013 showed that the proportion of individuals aged 60 years or older with at least one NCD was 76% in the overall population (15). Data from The Brazilian Longitudinal Study of Aging (ELSI-Brazil), a large-scale, nationally representative study of 9,412 participants aged 50 or older evidenced that 67.8% of these individuals presented ≥2 NCD and 47.1% ≥3 NCD, with an increase in the number of morbidities according to age (16, 17). It is important to consider that, in addition to being elderly; these patients with DM have an average of 15 years since diagnosis.

Patients with T1DM had a mean age of 35 years. When the morbidities most commonly related to T1DM are considered, we observe a higher prevalence of microvascular complications directly linked to DM, such as retinopathy, kidney disease and neuropathy, in addition to hypothyroidism. These findings are also in line with the literature that shows a higher presence of other autoimmune diseases, such as hypothyroidism (18). Regarding the morbidities associated with DM, the frequencies observed among patients with T1DM in the present study are similar to the prevalence found in a study with over 50,000 patients with T1DM in Europe and the United States (19). In this epidemiological study, prevalence of 14 to 25% of hypertension, 28 to 51% of dyslipidemia, 51 to 69% of overweight, and 20 to 33% of obesity were reported for patients with T1DM aged between 26 and 50 years old (19).

Regarding chronic complications, a higher prevalence of microvascular complications (retinopathy, blindness, microalbuminuria and kidney disease), diabetic foot and neuropathy was observed in the group of patients with T1DM, while macrovascular diseases (CVD, coronary disease, and cerebrovascular disease) and report of impotence were more frequent in those with T2DM. However, prevalence of lower limb amputation did not differ between groups. There is a paucity of studies that report the frequency of chronic complications in people with DM in Brazil. In a national, multicenter study that evaluated chronic complications in T2DM based on data from 2008 (8), the frequencies reported contrast with the ones found in the present study. Costa et al. reported a prevalence of diabetic foot of 1.1%, neuropathy of 27.7%, retinopathy of 42.4%, blindness of 2.9%, amputation of 4.7%, while in the current study these frequencies in T2DM were 2.0%, 9.7%, 11.2%, 1.0%, 1.0%, respectively. The current study, although not representative of the Brazilian population, offers an opportunity to describe the frequencies of micro- and macrovascular complications in a different scenario where most of T2DM were seen in private health care services.

Treatment for dyslipidemia reached a percentage of 97% in both T1DM and T2DM patients. This rate is surprisingly high when compared with the results of other studies. The ARATEUS study evaluated the medical charts of 662 patients with T2DM and observed that in the first 2 years of follow-up, only 29% of patients were in use of statins for the management of the dyslipidemia (20).

As expected, pharmacological treatment of T1DM consisted of insulin use in 100% of cases, accompanied by the utilization of certain classes of oral medication, among which biguanide was the most commonly used drug, having been available for the treatment of DM for a long time. ISGLT2 have been gaining space in the prescription of therapy for T1DM in this sample, where about 50% of patients were followed up in the private health system. Among patients with T2DM, a higher percentage of use of more recent drugs that are still not available in the public healthcare system (SUS, *Sistema Único de Saúde*) was observed during the study. This includes drugs such as iDPP4 (26.8%) and iSGLT2 (21.9%), which were being used in a frequency similar to that of sulfonylureas (31.4%). GLP1 analogues were being used by only 2.0% of patients with T2DM. This scenario must be interpreted in the light of the knowledge that 75% of the T2DM patients in the study sample were monitored in the private health system. It is interesting to note that 27.3% of patients with DM2 used insulin in this sample.

Regarding the glycemic target, the present study found that, among patients with T1DM, 25.3% had HbA1c <7% at the baseline visit and 22.5% at the end of the 2-year follow-up, and among the cases with T2DM, these prevalence rates were 45.5% and 51.0%, respectively. Population-based studies conducted in Brazil and involving the assessment of glycemic control in patients treated at the public health system showed that 26% of patients with T2DM were within the HbA1c <7% target (13), and 11.6% of adults and 23.2% of children and adolescents with T1DM reached this goal of HbA1c (14).

A multicenter study conducted in Latin America collected data on patients seen in the private health-care system, including 878 patients from Brazil, and found a result similar to the results presented here, with 40% of patients having HbA1c <7% (21). In a recent robust study evaluating patients with T1DM in the United States, Austria and Germany, in specialized DM care centers, a mean (± SD) of HbA1c of 8.1 ± 1.6% was observed in European centers and 8.6 ± 1.8% in American centers, with a percentage of patients who reached the HbA1c target <7% of 39% and 21%, respectively (19).

Importantly, the proportion of patients who did not achieve the individualized target in the present study were also alarmingly high (79% for T1DM and 53.3% for T2DM). The low proportion of patients with adequate glycemic control among both T1DM and T2DM patients is even more worrisome, when it is observed that there were no significant improvements in the mean HbA1c levels nor in the frequency of patients within the HbA1c target in both public and private sectors during the 2-year follow-up. This finding might indicate the existence of clinical inertia, that is, that situation in daily clinical practice in which the medical specialist is unaware or does not feel confident about the clinical condition of the patient and, as a result, tends to not adopt any correction of the therapeutic management in the face of unsatisfactory glycemic control. Clinical inertia is due to at least three factors: overestimation of the care provided, use of unfounded reasons to avoid intensification of therapy, and the lack of a well-trained interdisciplinary team to help the patient achieve the desired therapeutic goals (22). Better and faster results in glycemic control can only be achieved safely with educational strategies, structured self-monitoring of blood glucose and adequate pharmacological therapy in most cases (23).

Alongside glycemic treatment, adequate weight maintenance is another important factor. Obesity was observed in 22.2% and 40.7% of patients with T1DM and T2DM, respectively. During the follow-up, no significant weight loss was observed for both DM groups. Emphasizing the importance of weight loss for glycemic control in T2DM, a recently published study demonstrated reduced blood glucose and improved secretion and sensitivity to insulin in patients with DM and obesity undergoing weight loss, either through diet or surgery (gastric bypass). Moreover, after weight loss, there was a reduction in the dose of the antidiabetic medications by approximately 75% in both groups (24). In the Look AHEAD study, the behavioral management of obesity associated with lifestyle interventions (diet and physical activity) was superior to the DM education program in terms of weight loss and glycemic control in patients with DM and overweight. Furthermore, there were reductions in hospitalizations, medication use and health-related costs in the first group (25).

The current study has some limitations that should be considered when interpreting and extrapolating the data. The main limitation is that the sample population analyzed here is not representative of the Brazilian population. However, this lack of representativeness was expected at a certain level as the study was designed to assess the management and glycemic control of individuals with DM followed in clinical research centers that were selected and invited to participate from a pre-established list, not randomly chosen. Despite this, participating centers were located in cities of varied sizes and from different regions of Brazil, with some of them being part of the public health system (SUS). Although there was loss of follow-up during the study, the participants initially selected did not differ from those who remained in the study for the longitudinal analysis. This observation does not guarantee that participants who had missed follow-up visits had better or worse glycemic control during the follow-up. Despite these considerations, the results of glycemic control presented here are similar to the findings of other studies conducted in the Brazilian population with DM (11–14). Another limitation is the fact that the study sample is comprised of patients with higher level of education with the majority coming from private health-care facilities, which does not represent the Brazilian population. Regarding this, it is well-known that a higher prevalence of DM is observed among the population with a lower level of education (Vigitel, 2019) and that other social determinants, such as socioeconomic status and access to health services, are associated with worse glycemic control (26). In this scenario, the population profile of this study could also be seen as an opportunity for investigation, as studies involving the private health sector and individuals with higher education are scarce. Importantly, our results showed a poor glycemic control and features compatible with clinical inertia, even in a sample with this profile.

## Conclusion

Our study shows that after a 2-year period of follow-up, compared with baseline there were no relevant changes in the percentage of patients who achieved the goal of HbA1c <7%, in either T1DM or T2DM, in public or private health-care systems. These results might indicate that, besides the relevance of non-pharmacological treatment of diabetes, there is important therapeutic inertia that also needs to be addressed.

## Data availability statement

The raw data supporting the conclusions of this article will be made available by the authors, without undue reservation.

## Ethics statement

The studies involving human participants were reviewed and approved by The study was conducted after the approval by the ethics committee of the Universidade Federal de São Paulo (São Paulo, Brazil), and the study was conducted in accordance with the Declaration of Helsinki and the International Conference on Harmonization guidelines for Good Clinical Practice. Informed consent was obtained from all patients. CAAE: 52622115.9.000.5505. The patients/participants provided their written informed consent to participate in this study.

## Author contributions

BA-P: Data interpretation; Writing - original draft; Writing - review & editing. FE: Investigation; Visualization; Roles/Writing - original draft; Writing - review & editing. MP: Data curation; Methodology; Project administration; Supervision; Validation; Roles/Writing - original draft; Writing - review & editing. GF: Conceptualization; Data curation; Project administration; Resources; Supervision; Validation; Roles/Writing - original draft; Writing - review & editing. All authors contributed to the article and approve the submitted version.

## Funding

This study was supported by Sanofi. Editorial support in the preparation of this publication was provided by DENDRIX and paid for by Sanofi. The funder was not involved in the study design, collection, analysis, interpretation of data, the writing of this article or the decision to submit it for publication.

## Acknowledgments

We thank Felipe Lauand for conceptualization and data aquisition, Marina Szacher who was responsible for project administration, resources and supervision, Ana Truzzi for formal statistical analysis and conceptualization, and Maura Gonzaga for formal statistical analysis.

## Conflict of interest

FE has received financial support for clinical research from Sanofi, Lilly, Novo Nordisk, Amgen, Abbvie, and Bayer, and served as a speaker for Lilly, Sanofi, AstraZeneca, Novo Nordisk and Bayer. MP and GF are employees of Sanofi.

The remaining author declares that the research was conducted in the absence of any commercial or financial relationships that could be construed as a potential conflict of interest.

## Publisher’s note

All claims expressed in this article are solely those of the authors and do not necessarily represent those of their affiliated organizations, or those of the publisher, the editors and the reviewers. Any product that may be evaluated in this article, or claim that may be made by its manufacturer, is not guaranteed or endorsed by the publisher.
